# LiverCancerMarkerRIF: a liver cancer biomarker interactive curation system combining text mining and expert annotations

**DOI:** 10.1093/database/bau085

**Published:** 2014-08-27

**Authors:** Hong-Jie Dai, Johnny Chi-Yang Wu, Wei-San Lin, Aaron James F. Reyes, Mira Anne C. dela Rosa, Shabbir Syed-Abdul, Richard Tzong-Han Tsai, Wen-Lian Hsu

**Affiliations:** ^1^Graduate Institute of BioMedical Informatics, College of Medical Science and Technology, Taipei Medical University, 250 Wu-Xin Street, Taipei, Taiwan 110, Republic of China, ^2^Institute of Information Science, Academia Sinica, 128 Academia Road, Section 2, Nankang, Taipei, Taiwan 115, Republic of China, ^3^Institute of Chemistry, Academia Sinica, 128 Academia Road, Section 2, Nankang, Taipei, Taiwan 115, Republic of China and ^4^Department of Computer Science and Information Engineering, National Central University, 300, Jhongda Road, Jhongli City, Taoyuan County, Taiwan 320, Republic of China

## Abstract

Biomarkers are biomolecules in the human body that can indicate disease states and abnormal biological processes. Biomarkers are often used during clinical trials to identify patients with cancers. Although biomedical research related to biomarkers has increased over the years and substantial effort has been expended to obtain results in these studies, the specific results obtained often contain ambiguities, and the results might contradict each other. Therefore, the information gathered from these studies must be appropriately integrated and organized to facilitate experimentation on biomarkers. In this study, we used liver cancer as the target and developed a text-mining–based curation system named LiverCancerMarkerRIF, which allows users to retrieve biomarker-related narrations and curators to curate supporting evidence on liver cancer biomarkers directly while browsing PubMed. In contrast to most of the other curation tools that require curators to navigate away from PubMed and accommodate distinct user interfaces or Web sites to complete the curation process, our system provides a user-friendly method for accessing text-mining–aided information and a concise interface to assist curators while they remain at the PubMed Web site. Biomedical text-mining techniques are applied to automatically recognize biomedical concepts such as genes, microRNA, diseases and investigative technologies, which can be used to evaluate the potential of a certain gene as a biomarker. Through the participation in the BioCreative IV user-interactive task, we examined the feasibility of using this novel type of augmented browsing-based curation method, and collaborated with curators to curate biomarker evidential sentences related to liver cancer. The positive feedback received from curators indicates that the proposed method can be effectively used for curation. A publicly available online database containing all the aforementioned information has been constructed at http://btm.tmu.edu.tw/livercancermarkerrif in an attempt to facilitate biomarker-related studies.

**Database URL:** http://btm.tmu.edu.tw/LiverCancerMarkerRIF/

## Motivation and background

The National Cancer Institute at the National Institutes of Health, USA, defines a biomarker as any molecule in blood, tissue or body fluid that acts as an indicator of normal or abnormal biological processes, conditions or diseases (please refer to the definition at http://www. cancer.gov/dictionary?CdrID=45618). Specific gene biomarkers have been widely used in the diagnosis of cancer in high-risk patients, with the goal being to identify the most efficient therapeutic approach that can be used (1). However, uncovering clinically useful biomarkers from the results of laboratory research has proven to be a time- consuming process, which occasionally yields insufficient results in return for a the high expenditure of labor and funding (2). Biomarkers are validated using a process that typically includes three stages: laboratory validation, clinical trials and clinical application. Life scientists mainly conduct *in vitro* and *in vivo* experiments to examine the properties of candidate biomarkers. The final outcomes of these studies are affected by factors including the proficiency with which the researchers use experimental techniques, the quality of samples and the conditions of equipment and consumables. After preliminary laboratory studies provide promising results, the study is conducted at the next level, in which the previous procedures are repeated using patient samples and the consistency of outcomes is confirmed. Because the process of oncogenesis and its regulatory mechanisms within the human body are complex, not only the consistency might not be achieved but also the results obtained using mammalian animal models might contradict those obtained using patient samples. Furthermore, because cancers evolve over time, their overall gene-expression patterns change accordingly, which endows a stage-specific attribute to the biomarker examined. Lastly, after a biomarker has been approved using both laboratory and clinical studies, it is considered a potential target in cancer treatment. If the biomarker is a gene, drug design can be specialized toward manipulating and antagonizing the regulatory effects of the gene to facilitate the advancement of target therapies.

Discovering each biomarker can be a laborious process. However, the publications related to biomarker discovery often present highly ambiguous and contradictory results. Consequently, to enable life scientists to effectively use existing biomarker-related knowledge, we selected liver cancer as a target in an attempt to apply our text- mining–based approach in practice; we developed a curation system that allows curators to curate, view and edit descriptions of gene-related functions that are stored in our biomarker database. In this approach, text-mining techniques are used to automatically extract named entities including genes, microRNAs, diseases and other biomedical entities, such as investigative techniques applied in the laboratory-validation stage, that are often taken into consideration when assessing the feasibility of a biomarker. We named our text-mining–based curation system Liver Cancer bioMarker Reference Into Function (LiverCancerMarkerRIF) in view of theGeneRIF of the National Library of Medicine (NLM); theGeneRIF section of Entrez Gene contains narrative evidence on gene functions that are listed in publications. This section provides a platform that enables scientists to share and enrich gene-related functional annotations. The word ‘Gene’ is replaced with ‘Marker’ and this indicates the main purpose of our system: to enable searches for evidence in support of candidates of liver cancer biomarkers.

Using LiverCancerMarkerRIF, the curation process can be conducted directly on PubMed, and users are not required to navigate to independent Web sites. Curators can instantly edit and submit biomarker-related descriptions while browsing PubMed. General users can read the curated results that are extracted by combining the annotations of distinct curators. We consider this new process of curation process to represent a novel curation model that could encourage increased numbers of PubMed users to voluntarily contribute annotations to our system.

When the BioCreative IV user InterActiveTask (IAT) was being performed, curators were invited to install our system, and three of these curators (listed as the fourth, fifth and sixth authors of this article) performed the full curation task to classify evidential sentences on liver cancer biomarkers. All curated textual evidences were automatically stored on an online database together with hyperlinks to the original abstracts and Entrez Gene information in the PubMed database. Completing the task resulted in the generation of a database that contains evidential sentences that describe the relationship between biomarkers and liver cancer; this database is now available at http://btm.tmu.edu.tw/livercancermarkerrif. As of January 2014, a total of 208 candidate biomarker genes had been curated in the database.

## LiverCancerMarkerRIF curation system

**Figure 1.**
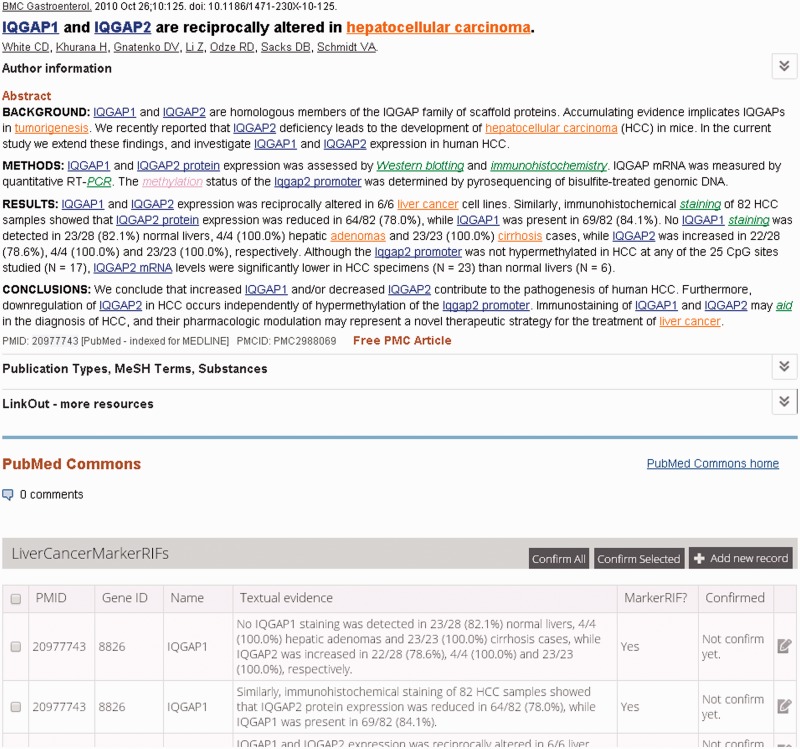
An augmented abstract on PubMed (PMID 20977743). The mentions of gene, disease, PTM and investigation techniques are highlighted in blue, brown, purple and green, respectively.

The LiverCancerMarkerRIF curation system was developed based on several advanced Web 2.0 technologies to provide the experience of augmented browsing (Augmented browsing refers to the experience of using a system that can automatically enhance or improve the information presented in Web pages.). The approach has been used increasingly on the Web to develop effective means to dynamically add Supplementary Information to a Web page without departing from the page (3). At its core, the approach relied on the use of asynchronous JavaScript and XML (AJAX), the framework of distinct Web browsers and Web services, which are described in detail in the next subsection. The functions of LiverCancerMarkerRIF include the recognition of genes, diseases, posttranslational modifications (PTMs), mutations, investigative techniques and the extraction of LiverCancerMarker-RIF evidential sentences. Figure 1 displays an augmented abstract on PubMed that was generated after installing LiverCancerMarkerRIF. The recognized named entities are highlighted using distinct colors that are coupled with hyperlinks to Entrez Gene and MeSH database pages containing additional detailed information. A table containing the evidential sentences curated from the abstract appears beneath the main body of the abstract.

**Figure 2.**
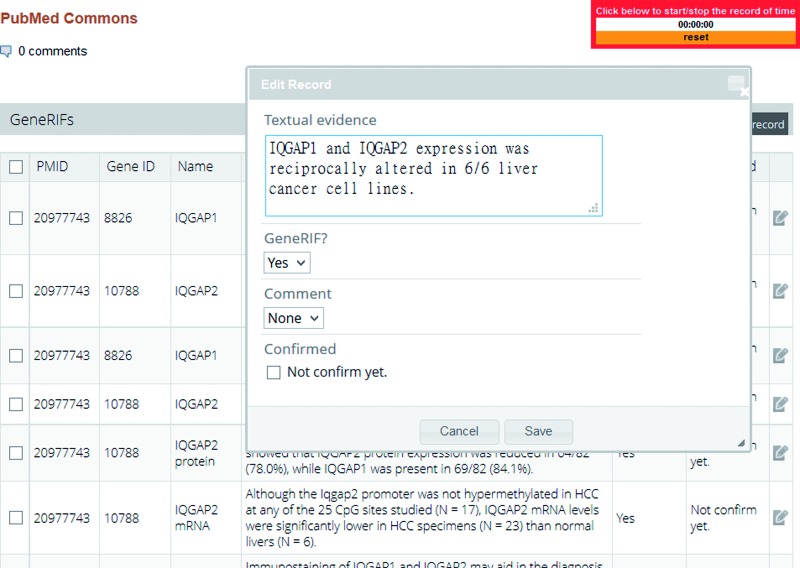
The curation interface of LiverCancerMarkerRIF.

When users log on LiverCancerMarkerRIF, a curation interface is made available (Figure 2) that curators can use to curate or modify extracted RIF sentences directly on PubMed. Once they are confirmed, the function-describing sentences are instantly submitted to our database and then can be accessed by other users.

### Main components of LiverCancerMarkerRIF

**Figure 3.**
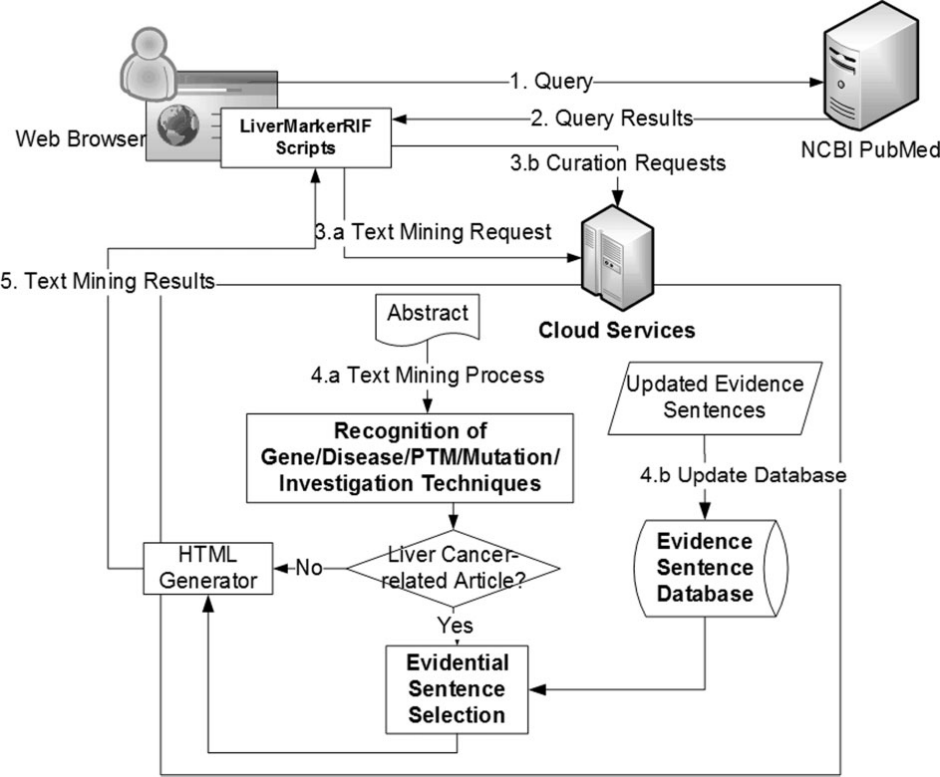
The system workflow of LiverCancerMarkerRIF. After installing LiverCancerMarkerRIF, it monitors the query sent to PubMed (step 1 and 2) and analyzes the returned results to extract the target text, such as the title and abstract. In step 3.a, the extracted text is sent to the cloud server to recognize biomedical entities. In step 4.a, in addition to recognizing entities, the curated evidential sentences for the article are further extracted from our database and combined with the entity recognition results if the text contained liver cancer-related terms. The results are finally sent back to the LiverCancerMarkerRIF in the client side to augment the original content. If some evidential sentences are curated by the curator, LiverCancerMarkerRIF will issue a curation request (step 3.b) and update our server-side database (step 4.b).

LiverCancerMarkerRIF consists of several text-mining components at its core that are deployed as Web services on the server side, and JavaScript scripts on the client side that work in-browser only when the user is on the PubMed Web site. An overview of our system is depicted in Figure 3. To use the system, a user must first install the LiverCancerMarkerRIF extension that can be downloaded from our Web site. For different Web browsers (Currently, LiverCancerMarkerRIF supports two browsers: Google Chrome and Mozilla Firefox), we use corresponding extension frameworks to implement the extension.

After LiverCancerMarkerRIF is installed, it intercepts the returned Web page when a user visits the PubMed Web site and invokes a query: the document object model (DOM; DOM defines the content, structure and style of an HTML document.) of the returned page is analyzed to identify the DOM element that contains the abstract text returned by PubMed. The raw text is then extracted from the element and sent to our cloud server, where the text is mined.

When the server receives the text-mining request, it forwards the request to our text-mining system. In our system, we have integrated five biomedical-concept name taggers. The first is a machine learning-based gene-mention tagger (4), which received an F-score of 0.862 on the BioCreative II gene-mention corpus (5). After a gene mention is recognized, a normalization subsystem links the identified gene names to their corresponding Entrez Gene IDs by using several normalization rules (6) and lexicons that contain genes and corresponding database identifiers collected from Entrez Gene and UniProt databases. The subsystem’s highest area under the precision/recall curve score was 0.435 in the BioCreative II.5 interaction normalization task (7) and it achieved an F-score of 0.687 on the Instance-Level Gene Normalization corpus (8).

The other five concept taggers are dictionary-based recognizers that function on the basis of the deterministic acyclic finite-state automaton. To identify microRNAs, the miRNA data published on the miRBase (http://www.mirbase.org/ftp.shtml) is used as the dictionary to recognize microRNAs mentioned in the abstract. For recognizing diseases and investigative technologies, we use the entire 2014 MeSH vocabulary (See http://www.nlm.nih.gov/mesh/filelist.html) as the source of the corresponding dictionaries to recognize related terms mentioned in the abstracts. The disease dictionary is constructed by collecting the terms under the MeSH tree number ‘C’, which denotes diseases. For example, in the case of the MeSH heading ‘Liver Neoplasms’ in the tree number ‘C04.588.274.623’, we collected the MeSH heading itself and all of the entry terms such as ‘Liver Cancer’ and ‘Neoplasms, Liver’ that are listed under the heading as the synonyms of the disease ‘Liver Neoplasms’. Furthermore, we applied the rules required to generate spelling variations. For instance, the variation ‘liver neoplasms’ was generated from the term ‘Neoplasms, Liver’. Lastly, following the recommendation of reviewers, disease names in Medic (9) were also integrated in our compiled dictionary. In the case of investigative technologies, the terms under the tree number ‘E05’, which denotes investigative techniques, were collected and compiled as a dictionary by following steps similar to the aforementioned steps. To recognize PTMs, we collected terms such as myristoylation and lipoylation from Wikipedia and several other sources, and to highlight mutation-related keywords, we used the following terms: mutation/mutant/mutate/mutated/mutations. After these dictionaries were compiled, each character that appears in each dictionary became a vertex that was used for generating directed acyclic word graphs, and the text was then matched with the generated graphs to recognize the corresponding entities.

After the recognition process is completed using the system, abstracts are checked to determine whether they contain terms related to liver cancer (the ‘Liver Cancer-related Article?’ step in Figure 3). We recurrently use the hierarchical structure of MeSH to confirm the existence of terms under the MeSH heading ‘Liver Neoplasms’. Abstracts that contain these terms are then processed to select the candidate evidential sentences. To collect distinct types of annotations from users, we list the sentences that contain recognized gene mentions as candidates, and allow users to classify these sentences into five predefined categories: relevant, irrelevant, negative, entity-recognition error and indefinite. After the results of the augmented search are returned to the user’s browser, the LiverCancerMarkerRIF extension can integrate the results into the displayed page.

## LiverCancerMarkerRIF in the BioCreative User IAT

**Figure 4.**
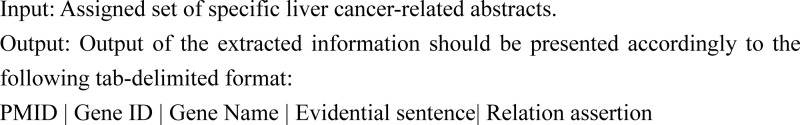
The details of the curation task protocol in BioCreative IV IAT.

The user IAT of BioCreative IV was designed to encourage text-mining teams to develop text-mining systems that can support a biocuration task (10). Through the participation in IAT, we recruited six biocurators, three of whom participated in the entire curation test (the fourth, fifth and sixth authors) that was designed to test the system by following the curation task protocol shown in Figure 4; the other three biocurators were involved in predefined short tasks of the IAT, which included tasks such as system installation and testing the functionality of the system, and we received these curators’ feedback regarding their first impressions of the system.

To perform the complete proposed curation task, the curators were provided a list of genes related to liver cancer, together with three sets of abstracts. The gene list contained potential liver cancer biomarkers, which were collected from several review articles (11–13). In the case of all candidate genes extracted from an abstract, the LiverCancerMarkerRIF extension arranges the ones that match the gene list ahead of other genes. The curation data set used for the complete curation task contained 190 abstracts that were retrieved from PubMed by using the following two query terms:

- (((blood[Title/Abstract] OR serum[Title/Abstract] OR urine[Title/Abstract]) AND clinical[Title/Abstract]) OR diagnosis[Title/Abstract]) AND liver cancer[Title/Abstract]
- (carcinoma, hepatocellular[MeSH Terms]) AND biomarker.

The first query is defined by domain experts when searching for information of interest within abstracts, and the second is a comparatively more straightforward query that is used to search for liver cancer biomarkers. The collected abstracts were divided into three sets in correspondence with the three curators who participated in the entire task, and these sets contained 63, 63 and 64 abstracts, respectively. Within the ∼1.5-month evaluation period, we asked each curator to perform complete manual curation on one set, and LiverCancerMarkerRIF-assisted curation on the other two sets. In the case of each data set, a curator was required to extract the following information: PubMed ID (PMID) of the abstract, gene terms and the corresponding gene ID from Entrez Gene, evidential sentences indicating that a gene is a biomarker for liver cancer, and relationship affirmation in the case of the tool-assisted curation (defined in the following section). Detailed scenarios of the two tasks are described next.

### Manual curation task

**Figure 5.**
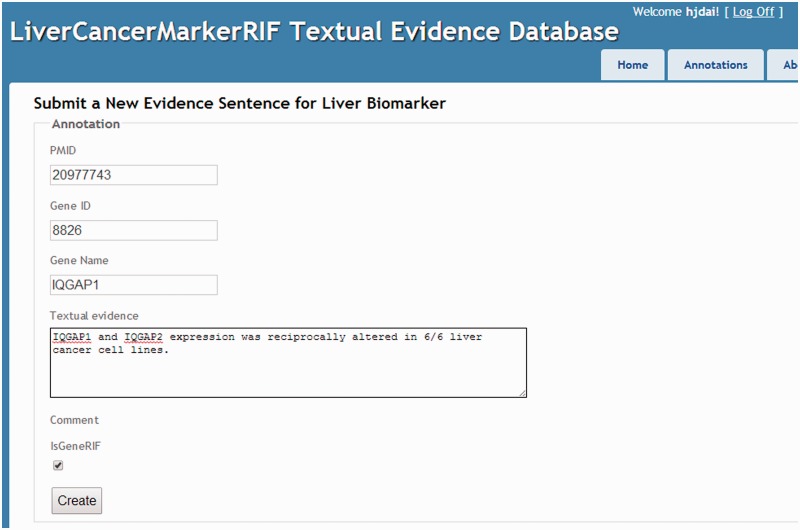
The manual curation interface.

Curators were assigned one set of PubMed abstracts and were required to submit their annotations manually to the database of LiverCancerMarkerRIF. To facilitate the submission process, we constructed a manual submission page (Figure 5).

### LiverCancerMarkerRIF-assisted curation task

In the case of the two other sets of abstracts, the curators extracted the information of interest with the assistance of LiverCancerMarkerRIF. A system tutorial featuring hands-on examples was provided to guide the curators. After the system was installed, the PubMed links containing the curation sets were sent to the curators through e-mail. They viewed the subjects of curation and text-mining–augmented results directly on PubMed through these links, and performed the curation tasks by using the curation interface of LiverCancerMarkerRIF.

Figure 2 shows the curation interface developed for IAT. All sentences in an abstract that contain gene names are extracted and sorted by LiverCancerMarkerRIF, and the suggested candidate sentences are listed below the abstract. Records of each sentence can be edited using the curation interface, which allows curators to add a new RIF sentence or modify the content of an existing textual sentence and validate whether the sentence conveys RIF knowledge. Furthermore, in the case of false-positive sentences, curators must confirm and assign them into four negative categories: irrelevant, negative, entity recognition error and indefinite. Table 1 displays an example selected from each category. An annotation guideline is also available at our Web site that remote curators can use as a reference (System Description/Curation Guideline section at http://btm.tmu.edu.tw/livercancermarkerrif/). Once a curator confirms and saves the results through the curation interface of LiverCancerMarkerRIF, the result is submitted and stored in the same database in which the curator’s results were submitted manually. Annotations provided by distinct curators are stored separately to allow them to be compared and analyzed.

**Table 1.** Examples for the five sentence categories and their corresponding curated sentences

Category	Gene candidate	Example sentence
Relevant	AFP-L3	**AFP-L3** is strongly correlated with the HCC patient outcome: a high level of **AFP-L3** is indicative of a poor patient prognosis.
Irrelevant	NGF	The serum levels of **NGF** were measured by enzyme-linked immunosorbent assay.
Negative	Cyclin D1	The expression of **Cyclin D1** was not correlated with CDK4 expression, tumor grades, survival rate, and any clinicopathological parameters.
Named entity recognition error	TACE	Aim: To assess the safety and efficacy of **TACE** in very elderly patients.
Indefinite	AFP-L3, DCP	The specificity of **AFP-L3** and **DCP** in the studied population was 78.5 and 100%, respectively.

The texts highlighted in bold indicate the gene candidates

In our system, a time meter is shown in the upper right-hand corner of the screen (Figure 2), which allows curators to log the time spent on curation. The curators who participated were asked to record the curation time required for both the manual and system-assisted curation tasks. After the tasks were completed, we were able to determine the utility of our tool by comparing the time spent by the curators on the two tasks. We initially planned to ask the curators to annotate the entire set of 190 abstracts collected from PubMed. However, limited by the time required to concurrently perform two tasks and the cost of remote communication through e-mail, we modified the original plan and asked the curators to curate only the first 30 abstracts of each set; thus, 90 abstracts were curated by each curator. Finally, with the help of IAT organizers, all participants provided feedback on the utility and usability of our system by completing a user survey, in which they offered suggestions for further improvements of our system.

## Results

### LiverCancerMarkerRIF results in BioCreative IV

We received the evaluation results regarding system competence from the organizers of the BioCreative IAT track. Metricsof the evaluation indicated that the curators were satisfied with the system while they were performing predefined tasks, and the curators also indicated posttesting satisfaction. Two of the short-task participants failed to install the system extension on Firefox, but all full-task participants finished the assigned tasks and their feedback (The official IAT survey is available at http://ir.cis.udel.edu/biocreative/survey2.html) was consistent and positive [Figure 4 of the official IAT summary paper (10)]. Analysis of the feedback suggested that compared with other similar systems, the participants indicated higher satisfaction with our system and considered that it was easier to use, exhibited greater power in completing the assigned curation tasks and was more flexible in the modes of use. The curators also considered the highlighting of biomedical concept names to be extremely helpful and the instructions of LiverCancerMarkerRIF to be simple and clear. All curators agreed that LiverCancerMarkerRIF can help in completing the curation tasks, and one of the curators strongly agreed with the view that using the system can lower the time required to complete the curation of liver cancer biomarkers.

**Figure 6.**
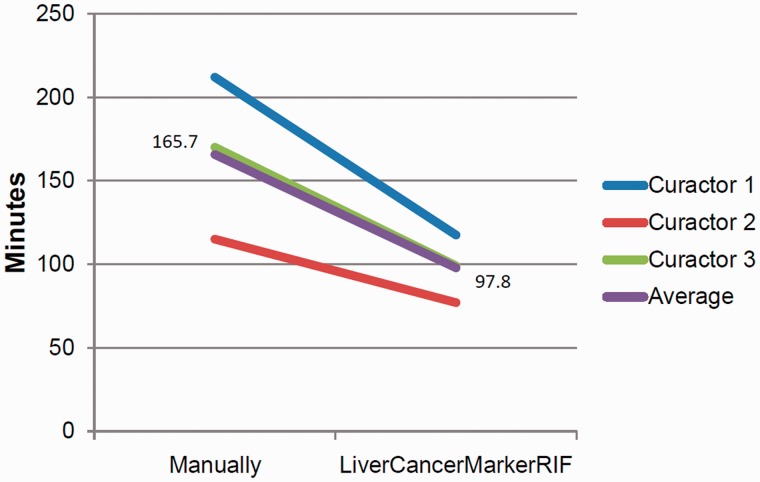
Curation time comparison.

In certain cases, curation time was higher when using LiverCancerMarkerRIF than manual curation; this occurred during the curation process when a curator confronted numerous irrelevant sentences and thus required a substantial amount of time to determine the negative class to which each of the sentences had to be assigned. During manual curation, such sentences were omitted, and this reduced the number of curations and thus the time required for curation. Figure 6 presents a comparison of the average time required for curating the 30 abstracts through manual and LiverCancerMarkerRIF-assisted curation, respectively. As curators may spend more time deciding the corresponding class for negative sentences during LiverCancerMarkerRIF-assisted curation, the average time for curating an abstract still dropped from 5.52 to 3.26 min when our system was used.

Currently, numerous curation tools are available to help curators in accomplishing specific curation tasks. However, most of these tools require curators to navigate away from PubMed and accommodate distinct user interfaces or Web sites to complete the curation process. By contrast, LiverCancerMarkerRIF provides a user-friendly method that can be used for accessing text-mining–aided information and a concise interface to assist curators while they remain at the PubMed Web site. We expect that this unique feature of our system will encourage increased numbers of PubMed users to voluntarily contribute their annotations to our database.

### Curation results

The feedback and the curated results indicated that the participating curators comprehended the notion and function of LiverCancerMarkerRIF. For instance, the sentences ‘Nowadays, **alpha fetoprotein** is the most widely used tumor marker for screening and diagnosis of hepatocellular carcinoma.’ and ‘**Annexin A2** was then selected for further verification.’ were deemed by all curators to be ‘Relevant’ and ‘Irrelevant’ in relation to the genes ‘AFP’ and ‘ANXA2’, respectively. The sentence ‘Between January 2003 and December 2005, we enrolled 115 treatment-naive patients who received **TACE** as an initial treatment modality.’ was marked as ‘Entity recognition error’ because trans-arterial chemo-embolization (TACE) is a treatment rather than a gene in this case. The sentence ‘Expression of BRM mRNA, but not **BRG1** mRNA, was significantly reduced in primary HCC tumours, compared to non-tumour tissue counterparts.’ was considered as a ‘Negation’ in relation to BRG1 because its expression remains unaffected in primary HCC tumors.

After the curators had completed the task, we calculated the value of Cohen’s kappa to determine the inter-annotator agreement by considering the curation task as a binary classification system used for selecting evidential sentences; the results are listed in Table 2. In terms of the comparison of kappa values between two curators, Curator C’s annotations appeared to be slightly different from those of the other two curators, possibly because of his medical background; by contrast, the other two annotators have a biochemistry background. The curation results generated could be ambiguous when the knowledge domains of curators are distinct; this is because the curators might inspect the candidate sentences from divergent perspectives such as, in this case, a clinical versus biochemical point of view. Furthermore, the negative categories, notably ‘Irrelevant’ and ‘Indefinite’, feature a certain amount of innate vagueness. An example is the case of the disagreement regarding the sentence ‘Serum **GP73** concentration was significantly correlated with the grading of fibrosis (r = 0.32, and 0.35, in 633 and 472 patients, respectively)’. In relation to GP73, this sentence might be considered ‘Irrelevant’ by one curator because it is a description of the correlation between GP73 concentration and fibrosis and not liver cancer. However, another curator might be aware that fibrosis often cooccurs with or leads to the onset of liver cancer, and that liver cancer is strongly correlated with GP73 concentration. Therefore, although the intended meaning is not stated clearly, a curator might consider this statement to be ‘Indefinite’ because no other category is more fitting in this case.

**Table 2.** Cohen's kappa for the three curators

Curator pair	Cohen's kappa value
Curator A versus Curator B	0.7111
Curator A versus Curator C	0.5333
Curator B versus Curator C	0.5111

As of January 2014, a total of 208 biomarker candidate genes had been curated in our database. The top five genes among these candidate biomarkers are AFP (alpha-fetoprotein), SALL4 (spalt-like transcription factor 4), ACE (angiotensin I converting enzyme) and GPC3 (glypican 3), and prothrombin which are sorted according to the total number of curated evidential papers and the total number of curated evidential sentences. A total of 108 abstracts contain evidential sentences, and each gene is featured on average in 0.4 evidential abstracts and 5.1 evidential sentences; 2836 confirmed evidentiary sentences are present, among which 1387 are affirmed as ‘Relevant’.

## Usage scenario of LiverCancerMarkerRIF

**Figure 7.**
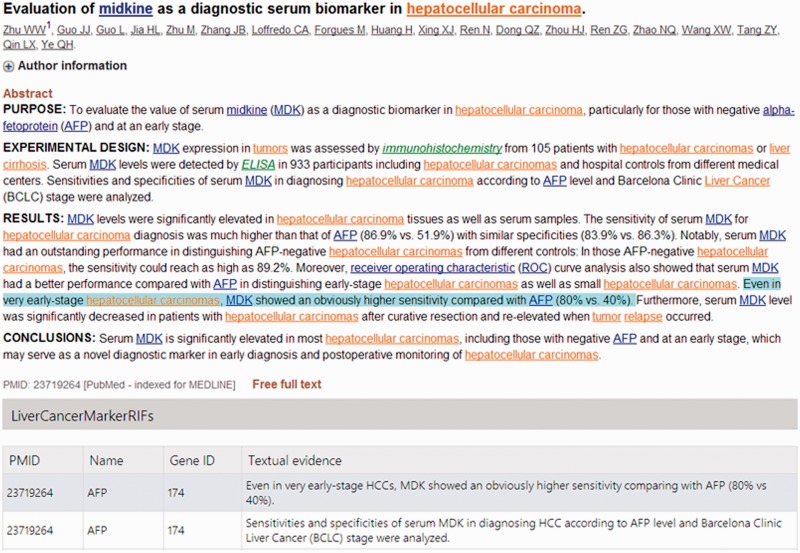
The curated table for non-logon users.

**Figure 8.**
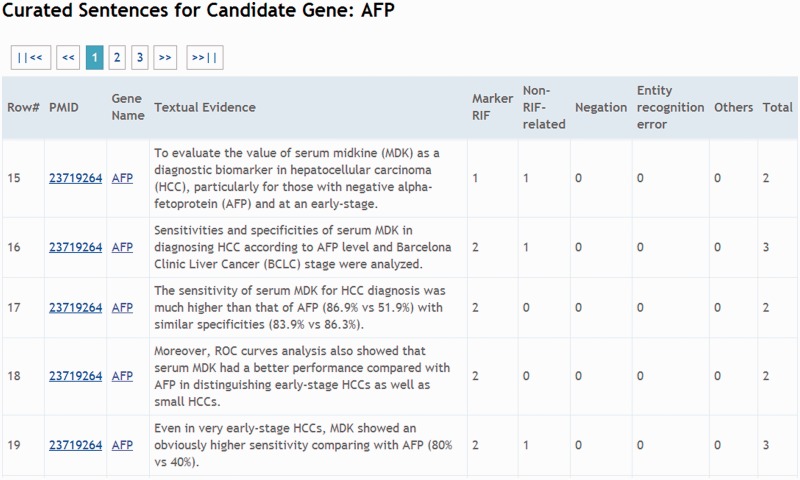
A snapshot of the curated affirmations for AFP from all curators.

After the IAT track, we have modified LiverCancerMarkerRIF to make it more convenient for online document curation. First, in contrast to the use of a small data set in the IAT track, we must now handle all types of possible abstracts that are curated and submitted from PubMed. Consequently, we have included a liver cancer-related article-filtering mechanism (Figure 3), which is described in the last paragraph of the ‘Main Components of LiverCancerMarkerRIF’ section. In the case of users who have installed LiverCancerMarkerRIF but have not yet logged on, the curation table will display existing curated results of the abstract if the article is related to liver cancer. If sentences feature multiple judgments, only those sentences in which the number of ‘Relevant’ votes overcomes the other options will be shown. Figure 7 presents an example of the voting scheme. A user who logs on to our database will notice that in the case of the first textual evidence shown in the curation table of Figure 7, two curators affirmed it as ‘Relevant’ and the third curator judged it as ‘Irrelevant’ (Figure 8, Row 19). Thus, the sentence remains in the curation table. By contrast, the curated evidence presented in Row 15 of Figure 8 is not shown in the curation table of LiverCancerMarkerRIF extension, because the majority vote was not ‘Relevant’ for this sentence (Note that Figure 7 does not show other evidence such as Row 17 and 18 of Figure 8 for concision. But they are listed in the curation table for the abstract.).

If a user logs on as a curator and an abstract retrieved by the user contains curated results, LiverCancerMarkerRIF will show, for reference, the voting results obtained for each candidate sentence in the five categories. Lastly, the time meter used in IAT has been removed from the interface, and because of privacy concerns and the problems confronted when logging onto a Google account, the original OAuth 2.0 authorization framework has been removed to protect the curators’ profile information.

## Perspectives

As indicated in the preceding section, analyzing the data set curated during IAT revealed that new curators might consider the definition of the four negative categories used for non-RIF sentences to be slightly unclear, and this could inconsistently distribute annotations, particularly between the ‘Irrelevant’ and ‘Indefinite’ categories. In the future, for each negative category, we will provide instructions that are more precise and detailed than those currently available, and we will also provide sample sentences in an effort to reduce ambiguity and improve inter-annotator agreement. The accumulated curated data set can be used as a corpus for developing an evidential-sentence classifier designed for liver cancer biomarkers.

All of the participating curators suggested that we focus only on specific sections within the abstract. In contrast to sections such as ‘Purpose’ and ‘Methods’, the ‘Results’ and ‘Conclusions’ sections often contain the main contribution of a paper. Therefore, focusing on ‘Results’ and ‘Conclusions’ can increase the efficiency of curation, and this will be considered during the development of the evidential-sentence classifier. To handle unstructured abstracts, a section categorizer that is based on our previous work (14) has been integrated into our system, which will be used to automatically divide abstracts into different sections including ‘Introduction’, ‘Methods’, ‘Results’ and ‘Conclusions’.

Lastly, regarding candidate evidential sentences, we have thus far examined only sentences that contain gene names but do not contain any negation terms. However, certain types of evidence are described using anaphora or cataphora. Therefore, we plan to include coreference resolution of pronouns to increase the comprehensiveness and abundance of the extracted biomarker information.

## References

[1] Easton D.F. Ford D. Bishop D.T. (1995) Breast and ovarian cancer incidence in BRCA1-mutation carriers. Breast cancer linkage consortium. Am. J. Hum. Genet., 56, 265–271 7825587 PMC1801337

[2] Rifai N. Gillette M.A. Carr S.A. (2006) Protein biomarker discovery and validation: the long and uncertain path to clinical utility. Nat. Biotech., 24, 971–983 10.1038/nbt1235 16900146

[3] Dai H.J. Tsai W.C. Tsai R.T.H. (2011) Enhancing search results with semantic annotation using augmented browsing. In: Proceedings of the Twenty-Second International Joint Conference on Artificial Intelligence (IJCAI11). Barcelona, Catalonia, Spain pp. 248–2423

[4] Tsai R.T.H. Sung C.L. Dai H.J. (2006) NERBio: using selected word conjunctions, term normalization, and global patterns to improve biomedical named entity recognition. BMC Bioinformatics, 7(Suppl. 5), S11 17254295 10.1186/1471-2105-7-S5-S11 PMC1764467

[5] Smith L. Tanabe L.K. Ando R.J.N. (2008) Overview of BioCreative II gene mention recognition. Genome Biol., 9(Suppl. 2), S2 18834493 10.1186/gb-2008-9-s2-s2 PMC2559986

[6] Dai H.J. Lai P.T. Tsai R.T.H. (2010) Multistage gene normalization and SVM-based ranking for protein interactor extraction in full-text articles. IEEE/ACM Trans. Comput. Biol. Bioinformatics, 7, 412–420 10.1109/TCBB.2010.45 20479501

[7] Krallinger M. Leitner F. Valencia A. (2009) The BioCreative II.5 challenge overview. In: Proceedings of the BioCreative II5 Workshop 2009 on Digital Annotations: 2009. Madrid, Spain, p. 19

[8] Dai H.J. Wu J.C.Y. Tsai R.T.H. (2013) Collective instance-level gene normalization on the IGN corpus. PLoS One, 8, e79517 24282506 10.1371/journal.pone.0079517 PMC3839972

[9] Davis A.P. Wiegers T.C. Rosenstein M.C. (2012) Medic: a practical disease vocabulary used at the comparative toxicogenomics database. Database **2012**, bar65 10.1093/database/bar065 PMC3308155 22434833

[10] Matis-Mitchell S. Roberts P. Tudor C.O. (2013) BioCreative IV interactive task. In: Proceedings of the Fourth BioCreative Challenge Evaluation Workshop October. Bethesda, MD, USA

[11] Masuzaki R. Karp S.J. Omata M. (2012) New serum markers of hepatocellular carcinoma. Semin. Oncol., 39, 434–439 22846860 10.1053/j.seminoncol.2012.05.009

[12] Behne T. Copur M.S. (2012) Biomarkers for hepatocellular carcinoma. Int. J. Hepatol., 2012, 859076 22655201 10.1155/2012/859076 PMC3357951

[13] Bertino G. Ardiri A. Malaguarnera M. (2012) Hepatocellualar carcinoma serum markers. Semin. Oncol., 39, 410–433 22846859 10.1053/j.seminoncol.2012.05.001

[14] Lin R.T.K. Dai H.J. Bow Y.Y. (2009) Using conditional random fields for result identification in biomedical abstracts. Integr. Comput. Aided Eng., 16, 339–352

